# LncRNA FAM30A as a potential biomarker associated with periodontitis and its role in inflammatory responses and osteogenesis

**DOI:** 10.1186/s12903-026-08033-y

**Published:** 2026-03-13

**Authors:** Yongbiao Huo, Limin Liu, Cancan Fan, Jieyi Chen, Haijing Gu

**Affiliations:** 1 Hospital of Stomatology, Sun Yat-sen University, 20th Floor, North Tower, Jinbin Tengyue Building, No. 49 Huaxia Road, Tianhe District, Guangzhou, 510000 China; 2 Guangdong Provincial Key Laboratory of Stomatology, Guangzhou, 510000 China

**Keywords:** Periodontitis, lncRNA, miR-28-5p, diagnostic, disease staging

## Abstract

**Background:**

This study investigated the expression patterns of lncRNA FAM30A in periodontitis, evaluated its diagnostic value, and elucidated the underlying molecular mechanisms.

**Methods:**

One hundred eight patients with periodontitis and 100 controls were enrolled. An in vitro model was established by stimulating human periodontal ligament cells (hPDLCs) with *Porphyromonas gingivalis (P. g)*-LPS. The expression levels of FAM30A in gingival crevicular fluid (GCF) and hPDLCs were quantified by RT-qPCR. ROC analysis assessed diagnostic accuracy, logistic regression pinpointed risk factors for stage III/IV periodontitis, and ELISA measured levels of inflammatory cytokines and MMP-1/MMP-3Cell viability and apoptosis were assessed by CCK-8 and flow cytometry. Osteogenic marker expression was quantified by RT-qPCR. The interaction among FAM30A, miR-28-5p, and KAT6A was confirmed using RIP and DLR assays.

**Results:**

The FAM30A levels rose significantly in GCF of periodontitis patients and in *P.g*-LPS-stimulated hPDLCs, while miR-28-5p expression dropped markedly. Elevated FAM30A improves periodontitis diagnosis (sensitivity 82.41%, specificity 96.00%). Its levels are higher in Stage III/IV periodontitis and independently predict periodontitis progression. Functionally, FAM30A knockdown attenuated *P.g*-LPS-induced inflammatory cytokine release, increased hPDLC apoptosis, suppressed osteogenic markers, and elevated MMP-1/MMP-3 secretion. These effects were partially reversed by miR-28-5p inhibition. Mechanistically, miR-28-5p targets FAM30A and KAT6A.

**Conclusion:**

The present study first demonstrates that FAM30A is a promising diagnostic biomarker, which is strongly linked to periodontitis staging (Stage I/II vs. Stage III/IV) and thus offers a new approach for clinical diagnosis. Additionally, silencing FAM30A may alleviate the progression of periodontitis by regulating the miR-28-5p/KAT6A axis, reducing inflammation, and promoting bone formation.

**Supplementary Information:**

The online version contains supplementary material available at 10.1186/s12903-026-08033-y.

## Background

Periodontitis is an inflammatory, infectious, and multifactorial disease that destroys periodontal tissues like alveolar bone, periodontal ligament, and root dentin [1]. Globally, 45–50% of people are affected, with 5–20% adult prevalence [2]. Severe cases number 750 million [3], costing $154.06 billion and €158.64 billion, respectively [4]. This disease typically presents with persistent bone resorption coupled with inflammatory cell infiltration, which eventually causes tooth mobility and loss. Yet, periodontitis is usually asymptomatic early on. By the time patients exhibit overt signs, the disease has usually progressed to an irreversible phase [5]. Crucially, periodontitis is closely linked to systemic diseases like coronary heart disease, stroke, and diabetes [6]. Hence, developing effective early diagnostic methods and taking timely actions are vital for slowing disease progression and improving outcomes.

Long non-coding RNAs (lncRNAs), which are over 200 nucleotides long and do not code for proteins, play a key role in cellular and tissue homeostasis [7]. Studies have identified HCP5 [8], NR_045147 [9], and MALAT1 [10] as lncRNA that drive periodontitis progression. Growing evidence suggests that stable and cost-effectively detectable lncRNAs, such as lncRNA HOTTIP [11] and NEAT1 [12], are potential diagnostic biomarkers for periodontitis. Family with sequence similarity 30 member A (FAM30A), a lncRNA also referred to as HSPC053, KIAA0125, and C14orf110, is situated on human chromosome 14q32.33 and consists of six exons. Prior research has highlighted its pivotal role in inflammatory disorders. For instance, a network analysis demonstrated that FAM30A is involved in the pathogenesis of lupus nephritis and could serve as a potential biomarker for liquid biopsy [13]. Moreover, FAM30A shows promise as a therapeutic candidate for rheumatoid arthritis [14]. Notably, Wu et al. detected an abnormal 2.33-fold increase in FAM30A levels by analyzing differentially expressed lncRNAs (DElncRNAs) in periodontitis [5]. Similarly, during the initial phase of our current study, through the utilization of the GSE10334 dataset, we observed a 2.01-fold rise in FAM30A expression among periodontitis patients. Nevertheless, the precise function and underlying mechanisms of FAM30A in periodontitis remain unclear.

Based on the aforementioned research findings, we propose that FAM30A may contribute to the progression of periodontitis. To explore this hypothesis, we recruited clinical patients, analyzed FAM30A expression patterns, and examined its potential clinical relevance. Additionally, we investigated the underlying mechanisms by which FAM30A influences periodontitis through loss-of-function and rescue assays using in vitro models. Our study aims to narrow the knowledge gap regarding the clinical significance and potential mechanisms of FAM30A in periodontitis, offering novel insights for the management of periodontitis patients.

## Materials and methods

### Data source and the identification of differentially expressed genes (DEGs)

The microarray dataset GSE10334 was sourced from the Gene Expression Omnibus (GEO). This dataset comprised 247 samples collected from 90 patients, including 183 diseased (periodontitis-affected gingival tissue) samples and 64 healthy control samples. We utilized the DESeq2 and Limma R software packages to conduct differential expression analysis of genes. For the GEO dataset, GEO2R was used to identify differentially expressed genes, including lncRNAs. Significant differential expression was determined by applying thresholds of |log2 (Fold change) | > 1.0 and -log10 (Adjusted P-value) > 10.

### Participants

Patients diagnosed with periodontitis who received treatment at the Hospital of Stomatology, Sun Yat-sen University between June 2022 and December 2024 were enrolled in this study. Inclusion criteria were as follows: (a) confirmed diagnostic of periodontitis with staging assignment in accordance with 2017 World Workshop on the Classification of Periodontal and Peri-Implant Disease and Conditions [15], with the diagnostic criteria consistently applied throughout the recruitment period; evidence of alveolar bone resorption was verified by oral X-ray or CT scan; (b) first-time diagnosis; (c) availability of complete clinical data; (d) age ≥ 18 years. The exclusion criteria were as follows: (a) comorbid endocrine disorders; (b) autoimmune conditions, such as rheumatoid arthritis or systemic lupus erythematosus; (c) comorbid malignancies; (d) recent periodontal treatment or oral trauma; (e) dental implants, cervical caries, or restorations in affected teeth; (f) other systemic chronic inflammatory diseases. Additionally, 100 periodontally healthy individuals were recruited as controls. These controls met the following criteria: no clinical signs of periodontal inflammation, intact periodontal tissues, no radiographic bone loss, probing depth (PD) ≤ 3 mm, clinical attachment loss (CAL) < 1 mm, bleeding on probing (BOP) ≤ 10%, no periodontitis-related tooth loss, and no history of periodontal disease [16, 17].

Approval for this study was granted by the Hospital of Stomatology, Sun Yat-sen University Medical Ethics Committee. All participants provided written informed consent, and all experimental protocols strictly adhered to the principles in the Declaration of Helsinki.

### Clinical information and gingival crevicular fluid (GCF) collection

Baseline clinical parameters, including age, gender, and body mass index (BMI), were recorded for all participants. After the collection of baseline data, each participant underwent a comprehensive periodontal examination. Two trained, calibrated periodontists performed double-blind clinical assessments using standardized probes to measure periodontal pocket depth, attachment loss, and other core indicators, with consistent protocols for GCF collection, storage, and testing.

For GSF sampling, four test points were selected per tooth: the buccal mesial, buccal distal, lingual mesial, and lingual distal surfaces. Before sampling, dental calculus, plaque, and soft debris were meticulously cleared away, and the tooth surfaces were gently dried with air. Subsequently, sterile filter paper strips were then inserted into the base of the periodontal pockets and left in place for 30 s. The strips were transferred to 0.2 mL Eppendorf tubes filled with phosphate-buffered saline (PBS), excluding any samples contaminated with blood. Tubes were vortexed for 1 h, and the supernatants were collected and stored at -20 °C for subsequent analysis. The fluorescent polymerase chain reaction technique was utilized to detect pathogenic bacterial infections, including *Porphyromonas gingivalis (P. g)*,* Tannerella denticola (T.d)* and *Aggregatibacter actinomycetemcomitans (A.a)*, in the GCFs of patients. A Williams periodontal probe was employed to assess key periodontal parameters in accordance with the 2017 World Workshop on the Classification of periodontal and Peri-Implant Diseases and Conditions: CAL (defined as the distance from the cementoenamel junction to the base of the periodontal pocket), PD (defined as the distance from the gingival margin to the pocket base), and BOP. Interdental alveolar bone loss (ABL) —the core radiographic indicator for periodontitis staging—was evaluated via imaging examinations, with the degree of alveolar bone resorption assessed concurrently. Additionally, the gingival index (GI) was used to assess gingival health based on changes in color and texture of the gingival tissues.

### Periodontitis staging stratification

Based on the 2017 World Workshop on the Classification of Periodontal and Peri-Implant Diseases and conditions [18, 19], patients with periodontitis were stratified into two groups accordance with the four-stage staging system (Stage I-IV): the Stage I/II group, defined by clinical CAL ≤ 4 mm, PD ≤ 5 mm, no tooth loss attributable to periodontitis, and interdental ABL < 33% of root length; and the Stage III/IV group, characterized by CAL ≥ 5 mm, either PD ≥ 6 mm or PD > 5 mm with measurable CAL, interdental ABL > 33% of the root length, and/or the presence of complexity indicators including tooth loss due to periodontitis, furcation involvement, and vertical bony defects.

### Cell culture and transfection

Human periodontal ligament cells (hPDLCs), obtained from the ScienCell Research Laboratories (catalog no. 2630, ScienCell, USA), were cultured in DMEM (catalog no. 11965-092, Gibco, USA) supplemented with 10% FBS (catalog no. 10438026, Gibco, USA) and 1% penicillin-streptomycin (catalog no, M4655-500mL, Sigma-Aldrich, USA) in a humidified incubator with 5%CO_2_ and 95% air. hPDLCs were treated with *P.g* lipopolysaccharide (*P.g*-LPS, catalog no. tlrl-ppglps. invivoGen, USA) at concentrations of 0, 1, and 10 µg/mL for 6, 12, and 24 h. Based on prior studies [20, 21], a concentration of 10 µg/mL *P.g*-LPS for 24 h was selected for induction.

### Cell Transfection

Small interfering RNA (siRNA) targeting FAM30A (si-FAM30A) and negative control si-NC were sourced from GenePharma, while miR-28-5p mimic (catalog no. miR10000085-1-5), miR-28-5p inhibitor (catalog no. miR20000085-1-5), mimic NC (catalog no. miR1N0000001-1-5), and inhibitor NC (catalog no. 2N0000001-1-5) were obtained from RiboBio (China). The sequences for siRNAs or oligonucleotides were as follows: si-FAM30A#1 (sense) 5’-UAAAUACAAAGACCAAAAGUU-3’, si-FAM30A#2 (sense) 5’-ACUAAUUGAGAUGAUACACAU-3’, si-FAM30A#3 (sense) 5’-ACAUAGAGUGUCUUCUUUCCA-3’, si-NC (sense) 5’-UUCUCCGAACGUGUCACGUTT-3’; miR-28-5p mimic (sense) 5’-AAGGAGCUCACAGUCUAUUGAG-3’ and miR-28-5p mimics (anti-sense) 3’-CAAUAAGACUGUGAGCUCCUUU-5’, mimic NC (sense) 5’-UUCUCCGAACGUGUCACGUTT-3’ and mimic NC (anti-sense) 3’-ACGUGACACGUUCGGAGAATT-5’, miR-28-5p inhibitor (sense) 5’-CUCAAUAGACUGUAGCUCCUU-3’, inhibitor NC (sense) 5’-CAGUACUUUUGUGUAGUACAA-3’. These siRNAs or oligonucleotides were complexed with Lipofectamine 3000 transfection reagent (catalog no. 1656200, Invitrogen, USA) and added to the cells. The culture medium was replaced after 8 h.

### Osteoblastic differentiation of hPDLCs

To promote osteoblast differentiation, hPDLCs were seeded at a density of 1 × 10^5^ cells per well in 12-well plates and cultured in osteogenic medium for 1, 3, 7, and 14 days. This medium comprised 90% DMEM supplemented with 10% FBS, 100 nmol/L dexamethasone (catalog no. D4902-25MG, Sigma-Aldrich, USA), 10 mmol/L β-glycerophosphate (catalog no. G6501, Sigma-Aldrich, USA), and 50 µg/mL ascorbic acid (catalog no. G9422-10G, Sigma-Aldrich, USA). The medium was refreshed every 2–3 days. Moreover, at day 7 of osteogenic differentiation induction, we assessed both the mRNA and protein expression of osteogenic markers and measured alkaline phosphatase (ALP) activity.

### RNA extraction and Real-time quantitative reverse transcription PCR (RT-qPCR)

Total RNA was isolated from GSF samples and hPDLCs using TRIzol reagent (catalog no. D312, Takara, Japan) and dissolved in RNAase-free solution. The purity of the RNA was confirmed by spectrophotometry, with A260/A280 ratios between 1.8 and 2.0 indicating high quality, suitable for subsequent cDNA synthesis. For reverse transcription, 500 ng of RNA was converted into cDNA using either the Prime Script RT Master Mix (for gene amplification; catalog no. RR047A, Takara) or the Mir-X miRNA First-Strand synthesis Kit (for miRNA amplification, catalog no. 638315, Takara, Japan). The cDNA was then quantified and amplified in triplicate using the SYBR Premix Ex Taq Kit (catalog no. RR420A, Takara, Japan) and miRcute Plus miRNA qPCR Detection kit (catalog no. FP411, TIANGEN Biotech, China), with primers on the ABI7300 real-time fluorescence quantitative PCR system. Primer sequences used are shown in supplementary Table S1. Gene expression levels of FAM30A, OCN, OPN, RUNX2, and BMP2 were normalized by GAPDH. U6 acted as an endogenous control for miR-28-5p. The relative gene expression was measured by the 2^−ΔΔCt^ method.

A sample is considered positive for the target bacteria only if it simultaneously meets the following conditions: (a) Detection of a specific fluorescent signal during amplification (a single peak on the SYBR Green melting curve with on non-specific banding); (b) Cycle threshold ≤ 35; (c) No fluorescent signal amplification in the negative control (using sterile double-distilled water instead of DNA template) ruling out contamination interference.

### Western blot

Cells were harvested and lysed in RIPA buffer (catalog no. P0013B, Beyotime Biotechnology, China) supplemented with protease and phosphatase inhibitors (catalog no. 5872, Cell Signaling Technology, USA). The lysate was incubated on ice for 20 min, followed by centrifugation to collect supernatant. Total protein concentration was quantified using the BCA protein assay kit (catalog no. P0012S, Beyotime Biotechnology, China). Protein samples were prepared by mixing with SDS-PAGE loading buffer and boiling for 5 min. Denatured proteins (30 µg) were loaded onto a 10% SDS-PAGE gel. Electrophoresis was performed at 80 V for 30 min, followed by separation at 100 V for 60 min. Proteins were transferred to a PVDF membrane via wet transfer at 90 V for 100 min. The membrane was blocked with 5% nonfat milk at room temperature (catalog no. 16370-1-AP, Proteintech, USA) for 2 h. The primary antibodies anti-OCN (catalog no. ab93876, Abcam; 1: 1000), anti-OPN (catalog no. ab8488, Abcam; 1:1000), anti-RUNX2 (catalog no. ab76956, Abcam; 1:1000), BMP2 (catalog no. ab284387, Abcam, 1:1000), anti-GAPDH (catalog no. ab9485, Abcam; 1:2500, USA). The PVDF membrane was incubated with the primary antibody overnight at 4℃, followed by incubation with an HRP-conjugated secondary antibody (catalog no. 7076, Cell Signaling Technology, USA) at room temperature for 1 h. After exposure to Beyo ECL Plus solution (catalog no. P0018S, Beyotime, China), the membrane was visualized using the Syngene Gene Genius gel imaging system. Protein band intensities were quantified using Image J software. The full uncut original pictures are shown in Supplementary Figures S1 and S2.

### Cell viability assay

Cell viability was assessed using the Cell Counting Kit 8 (CCK-8) assay (catalog no. CK04, Dojindo Molecular Technologies, Japan). Transfected cells were seeded into 96-well plates at a density of 5 × 10^3^ cells/100 µL. At 0, 24, 48, and 72 h, the culture medium was replaced with a mixture of 10 µL CCK-8 reagent and 90 µL DMEM, followed by incubation at 37℃ for 2 h. Finally, the optical density (OD) at 450 nm was measured using a microplate reader.

### Cell apoptosis assay

Apoptosis rate was evaluated using Flow Cytometry in conjunction with an apoptosis detection kit (catalog no. CA1020-20, Solarbio, China). Transfected cells were rinsed with pre-chilled PBS, then digested with trypsin and resuspended in 1×binding buffer. Next, 10 µL of Annexin V-FITC solution and 5 µL of PI solution were added to the cell suspension. After a 15-minute dark incubation, 300 µL of binding buffer was added, and the apoptosis rate was subsequently analyzed via flow cytometry.

### Enzyme-linked immunosorbent assay (ELISA)

Supernatants were collected from PDLCs. The concentrations of inflammatory markers, including IL-6, IL-8, and TNF, in both cell supernatants and GCFs will be determined based on the instructions provided by the kit manufacturer (human IL-6 ELISA kit [catalog no. E-EL-H6156]; human IL-8 ELISA kit [catalog no. E-EL-H6008]; human TNF-α ELISA kit [catalog no. E-EL-H0109, Elabscience, China]). Additionally, the expression levels of Matrix Metalloproteinase 3 (MMP-3, catalog no. E-EL-H1446, Elabscience, China) and MMP-1 (catalog no. E-EL-H6073, Elabscience, China) in the cell supernatant will also be assessed. For sample preparation, the cell supernatant was centrifuged at 1000 g for 20 min to remove impurities and debris, and then PMSF protease inhibitor was added. In the assay, 100 µL of sample was added to each well and incubated at 37℃ for 90 min. After the sample was removed, 100 µL of biotinylated antibody solution was added, and the mixture was incubated at 37℃ for 1 h. The wells were emptied and washed three times with 350 µL of solution each time. Then, 100 µL of HRP enzyme conjugate was added and incubated for 30 min. Substrate was added, the plate was incubated in the dark for 15 min. Subsequently, 50 µL of stop solution was added. After a final 15-minute wait, the OD at 450 nm was measured using a microplate reader.

### ALP activity assay

hPDLCs were induced to undergo osteogenic differentiation by culturing in osteogenic medium for 7 days. This medium comprised 90% DMEM supplemented with 10% FBS, 100 nmol/L dexamethasone, 10 mmol/L β-glycerophosphate, and 50 µg/mL ascorbic acid. After washing with PBS, the cells were lysed using 1% Triton and centrifuged at 10,000 rpm for 10 min. The absorbance related to ALP activity was then measured at 510 nm using an ALP detection assay kit (catalog no. BC2140, Solarbio, China).

### Nuclear-cytoplasmic fractionation assay

Additionally, the PARIS™ kit (catalog no. AM1921, Life Technologies, China) was utilized for nucleus-cytoplasm fractionation. Specifically, 5 × 10⁶ cells were resuspended in a lysis buffer for separating cytoplasmic and nuclear components and lysed on ice for 10 min, followed by centrifugation at 11,000 rpm for 10 min at 4℃. The supernatant was collected for cytoplasmic RNA extraction. The remaining pellet was processed further, and the cytoplasmic RNA from the pellet was isolated by resuspending it in an ice-cold cell disruption buffer. Subsequently, both cytoplasmic RNAs were eluted using an appropriate elution buffer. RT-qPCR was then conducted to assess the enrichment of FAM30A in the cytoplasm and nucleus, with GAPDH serving as the endogenous reference for the cytoplasmic fraction and U6 for the nuclear fraction.

### Bioinformatics analysis

The LncLocator database was employed to predict the subcellular localization of FAM30A. LncBook and DIANA databases independently predicted target miRNAs for FAM30A. We then analyzed the overlapping target miRNAs from both databases, identifying miR-28-5p among them. The ENCORI, miRDB, and microT_interaction databases were employed to predict target genes of miR-28-5p. The Venn platform was then used to identify overlapping target genes among the three datasets. Meanwhile, the STRING database constructed a protein-protein interaction (PPI) network and pinpointed the top 10 hub genes. Finally, KEGG pathway enrichment analysis of the overlapping targets was conducted and visualized via SRplot.

### Dual-luciferase reporter (DLR) assay

FAM30A or lysine acetyltransferase 6 A (KAT6A) fragments harboring either wild-type or mutant miR-28-5p binding sites were cloned into pGL3 vectors, generating wild-type (FAM30A-WT or KAT6A-WT) and mutant (FAM30A-Mut or KAT6A-Mut) luciferase reporter vectors, respectively. These vectors, along with NC inhibitor, NC mimic, miR-28-5p mimic, and miR-28-5p inhibitor, were transfected into PDLCs using Lipofectamine 3000. After 48 h, luciferase activity was measured using the Dual-luciferase reporter gene assay kit (catalog no. RG027, Beyotime Biotechnology, China).

### RNA Immunoprecipitation (RIP) assay

The interaction between FAM30A, miR-28-5p, and KAT6A was confirmed using the EZ-Magna RIP assay (catalog no. 17–701, Millipore, USA). In brief, hPDLCs were lysed in RIP lysis buffer containing magnetic beads conjugated with antibodies against Ago2 (for target enrichment) and IgG (as a negative control). The lysates were incubated overnight at 4℃. The resulting complexes were treated with proteinase K to digest proteins, followed by separate RNA isolation for subsequent RT-qPCR analysis.

### Statistical analysis

Data visualization and statistical analysis were performed using SPSS 23.0 and GraphPad Prism 9.0. Count data, which represent the number of occurrences in different categories, were expressed as n (%) and analyzed using the chi-square test. Measurement data, which followed a normal distribution, were presented as mean ± standard deviation (SD). The independent samples t-test was used for comparisons between two groups when the data met the assumptions of normality and homogeneity of variance. For multiple comparisons among more than two groups, one-way analysis of variance (ANOVA) followed by post hoc Tukey analysis was conducted. Pearson correlation analysis assessed the linear relationship between FAM30A and miR-28-5p. The receiver operating characteristic (ROC) curve evaluated the diagnostic and predictive performance of FAM30A levels for periodontitis and stage III/IV periodontitis. Logistic regression analysis was employed to identify potential risk factors for the development of stage III/IV periodontitis. A P value < 0.05 was considered statistically significant.

## Results

### Gingival crevicular fluid LncRNA FAM30A was notably upregulated in patients with periodontitis

**Fig. 1:**
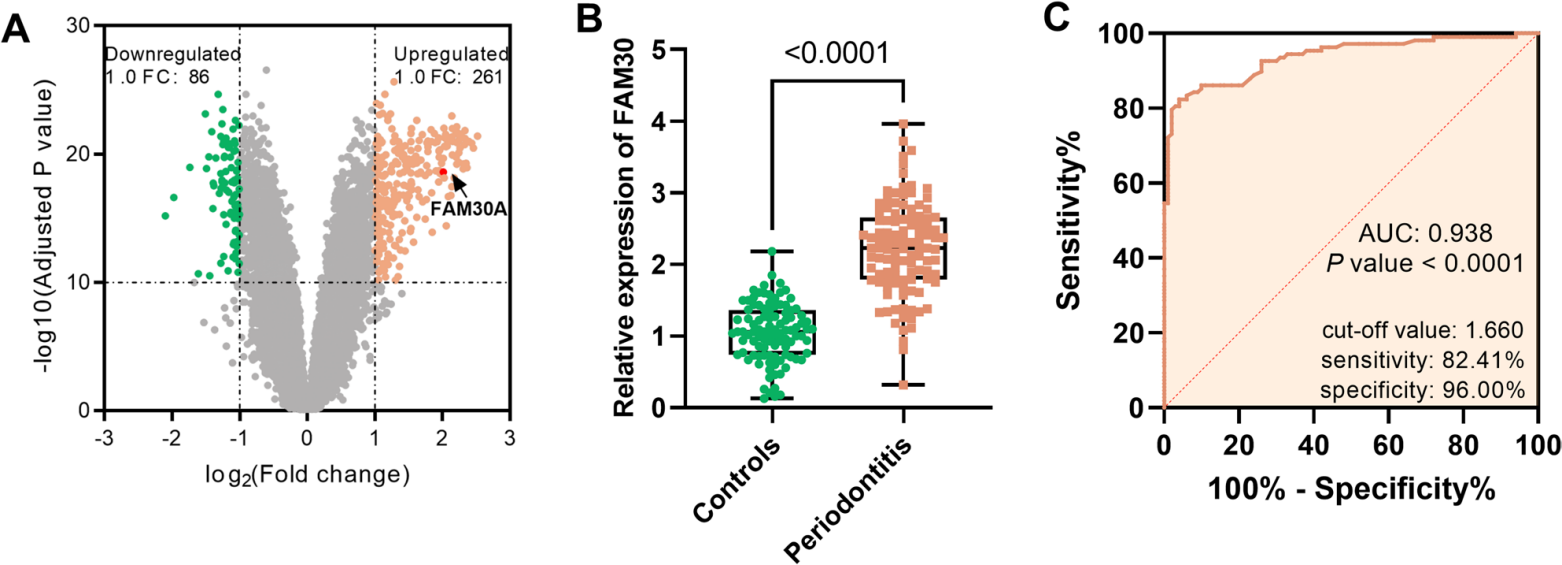
Gingival crevicular fluid (GCF) FAM30A levels and diagnostic significance. **A** A volcano plot presents differentially expressed genes in GSE10334, comparing periodontitis vs. healthy gingival tissues, including lncRNA FAM30A. **B** RT-qPCR FAM30A expression in GCFs of periodontitis patients and healthy controls. **C** ROC curves assess GCF FAM30A’s diagnostic ability to distinguish periodontitis patients from healthy ones. ****P < 0.0001

The GSE10334 dataset revealed 261 upregulated and 86 downregulated genes in periodontitis, with lncRNA FAM30A showing a 2.01-fold increase (Fig. 1A). To investigate FAM30A’s role in periodontitis, we recruited 108 patients with periodontitis and 100 controls, matched for demographics (age, gender, BMI) and lifestyle (smoking, drinking, diet, toothbrushing, *P* > 0.05, Table 1). Patients with periodontitis exhibited a higher detection rate and bacterial loads of three prevalent periodontal pathogens (*P.g*,* T.d*, and *A.a*) compared with the controls. They also showed higher levels of periodontal markers, including CAL, PD, ABL, BOP, GI, and PI (*P* < 0.05, Table 1). Inflammatory factors IL-6, IL-8, and TNF were also higher in patients with periodontitis than in the controls (*P* < 0.05, Table 1). Notably, GCF levels of FAM30A were elevated in patients with periodontitis than in the controls (*P* < 0.001, Fig. 1B).

**Table 1:** Clinical baseline characteristics of subjects

Indicators	Controls (*n* = 100)	Periodontitis (*n* = 108)	*P* value
Demographics			
Age (years)	46.07 ± 13.43	49.00 ± 12.96	0.112
Male (*n*, %)	51 (45.00)	64 (56.00)	0.265
BMI (kg/m^2^)	24.02 ± 3.70	24.26 ± 3.22	0.624
Lifestyle Factors			
Smoking (*n*, %)	59 (59.00)	67 (59.26)	0.672
Drinking (*n*, %)	56 (56.00)	57 (62.04)	0.677
Heavy-flavor diet tendency (*n*, %)	45 (45.00)	56 (51.85)	0.335
Toothbrushing ≥ 2 (times/day)	68 (68.00)	67 (62.04)	0.386
Periodontal pathogens			
* P.g* (*n*, %)	13 (13.00)	87 (80.56)	0.000
* T.d* (*n*, %)	3 (3.00)	73 (67.59)	0.000
* A.a* (*n*, %)	7 (7.00)	59 (54.63)	0.000
Periodontal indicators			
CAL (mm)	0.68 ± 0.27	4.00 ± 1.46	0.000
PD (mm)	2.22 ± 0.62	5.00 ± 1.22	0.000
ABL (%)	0.28 ± 0.10	28.63 ± 11.44	0.000
BOP (%)	6.53 ± 2.29	49.23 ± 9.55	0.000
GI	0.41 ± 0.13	2.00 ± 0.62	0.000
PI	0.62 ± 0.19	2.25 ± 0.74	0.000
Inflammatory Indicators			
IL-6 (pg/mL)	162.97 ± 49.45	422.79 ± 84.54	0.000
IL-8 (pg/mL)	131.89 ± 34.48	575.08 ± 99.70	0.000
TNF (pg/mL)	183.10 ± 55.94	583.97 ± 154.43	0.000

*Abbreviations BMI* body mass index, *PD* probing pocket depth, *CAL* clinical attachment level, *GI* gingival index, *PI* plaque index, *ABL* alveolar bone resorption length, *BOP* bleeding in probing, *IL-6* interleukin-6, *IL-8* interleukin-8, *TNF* tumor necrosis factor, *P.g Porphyromonas gingivalis, T.d Tannerella denticola, A.a*,* Aggregatibacter actinomycetemcomitans*

### LncRNA FAM30A overexpression holds promise as a diagnostic biomarker for periodontitis

Based on the mean serum FAM30A level (2.22 ± 0.65), periodontitis patients were split into high-(*n* = 57) and low- (*n* = 51) expression groups. Further grouping by mean clinical pathological features and pathogenic bacteria identification (binary) highlighted indicator trends and periodontal healthy gaps. As presented in Table 2, patients with high FAM30A expression not only had a higher detection rate of pathogenic organisms but also suffered from more stage III/IV periodontal conditions (*P* < 0.05). Specifically, they showed significantly longer PD (*P* < 0.0001), higher BOP scores (*P* = 0.002), greater CAL (*P* < 0.0001), more pronounced ABL (*P* = 0.011), and increased PI (*P* = 0.007) compared to their low-expressing group. Furthermore, patients with periodontitis exhibiting high FAM30A expression showed elevated GCF levels of IL-6 (*P* = 0.022), IL-8 (*P* = 0.012), and TNF (*P* = 0.036). Moreover, at a cut-off value of 1.660, FAM30A exhibited a sensitivity of 82.41% and a specificity of 96.00% in distinguishing patients with periodontitis from periodontally healthy individuals with an AUC of 0.938 (95%CI 0.9055–0.9706, *P* < 0.0001, Fig. 1C).

**Table 2:** Association of gingival crevicular fluid lncRNA FAM30 levels with clinical parameters in periodontitis

Indicators	Total (*n* = 108)	LncRNA FAM30 expression		*P* value
		Low (*n* = 51)	High (*n* = 57)	
*P.g*				
Negative	21	15	6	0.016
Positive	87	36	51	
*T.d*				
Negative	35	22	13	0.039
Positive	73	29	44	
*A.a*				
Negative	49	29	20	0.033
Positive	59	22	37	
CAL (mm)				
< 4.00	66	46	20	0.000
≥ 4.00	42	5	37	
PD (mm)				
< 5.00	66	45	21	0.000
≥ 5.00	42	6	36	
ABL (%)				
< 28.63	62	36	26	0.011
≥ 28.63	46	15	31	
BOP (%)				
< 49.23	52	31	21	0.020
≥ 49.23	56	20	36	
GI				
< 2.00	55	31	24	0.057
≥ 2.00	53	20	33	
PI				
< 2.25	52	32	20	0.007
≥ 2.25	56	19	37	
IL-6 (pg/mL)				
< 422.79	55	32	23	0.022
≥ 422.79	53	19	34	
IL-8 (pg/mL)				
< 575.08	49	30	19	0.012
≥ 575.08	59	21	38	
TNF (pg/mL)				
< 582.97	56	32	24	0.036
≥ 582.97	52	19	33	

*Abbreviations CAL* clinical attachment level, *GI* gingival index, *PI* plaque index, *ABL* alveolar bone resorption length, *BOP* bleeding in probing, *IL-6* interleukin-6, *IL-8* interleukin-8, *TNF* tumor necrosis factor. *P.g Porphyromonas gingivalis, T.d Tannerella denticola, A.a*,* Aggregatibacter actinomycetemcomitans*

### FAM30A and periodontitis staging association

**Fig. 2:**
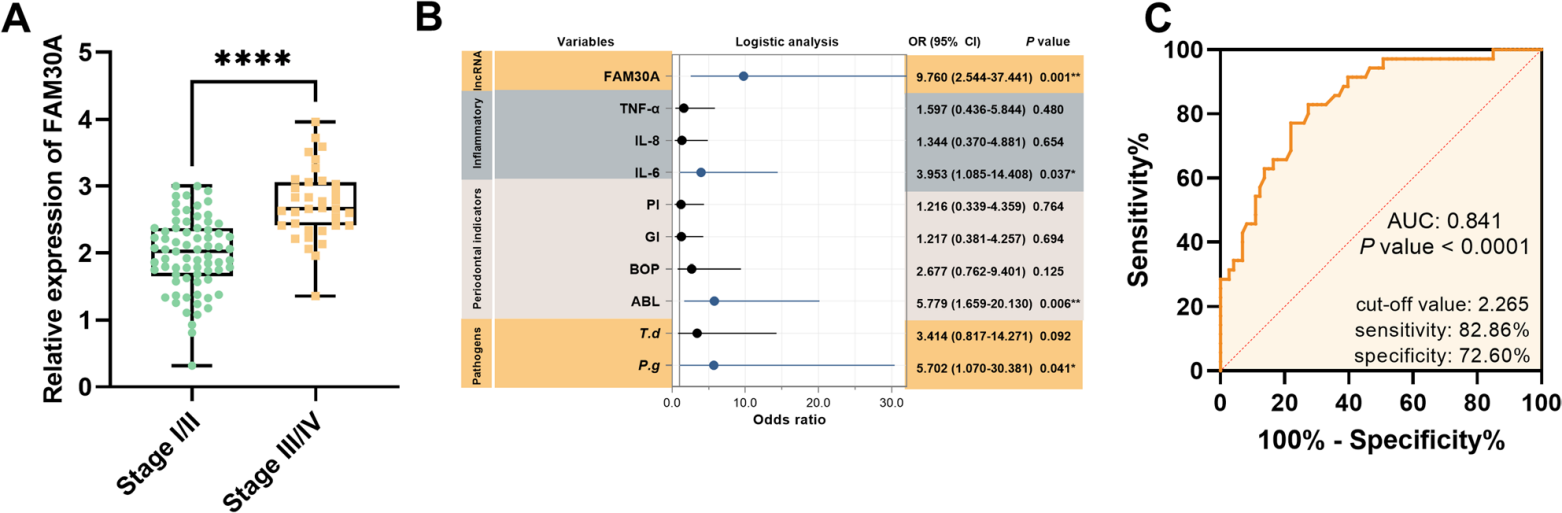
FAM30A levels predict stage III/IV periodontitis. **A** RT-qPCR measured FAM30A in the GCF from patient stage I/II and stage III/IV periodontitis. **B** Forest plots illustrate potential risk factors for stage III/IV periodontitis. **C** ROC curves evaluate FMA30A’s predictive power for stage III/IV periodontitis. ****P < 0.0001

To delve deeper into the relationship between FAM30A and periodontitis staging, patients with periodontitis were stratified into stage I/II and stage III/IV groups based on their disease status. Baseline clinical characteristics were then compared between these two groups. The stage III/IV group exhibited a significantly higher detection rate of *P.g* and *T.d*. Additionally, this group presented with more pronounced periodontal clinical indicators and elevated inflammatory factors levels (*P* < 0.05, Table 3). Notably, FAM30A levels were also significantly higher in the stage III/IV group than in the stage I/II group (*P* < 0.001, Fig. 2A).

**Table 3:** Clinical baseline characteristics of periodontitis patients stratified by disease staging

Indicators	Stage I/II group (*n* = 73)	Stage III/IV group (*n* = 35)	*P* value
Demographics			
Age (years)	48.21 ± 13.98	50.75 ± 10.44	0.112
Male (*n*, %)	46 (63.01)	18 (51.43)	0.298
BMI (kg/m^2^)	24.48 ± 3.29	23.77 ± 3.06	0.624
Lifestyle Factors			
Smoking (*n*, %)	49 (67.12)	18 (51.43)	0.140
Drinking (*n*, %)	40 (54.79)	17 (48.57)	0.681
Heavy-flavor diet tendency (*n*, %)	36 (49.32)	20 (57.14)	0.538
Toothbrushing ≥ 2 (times/day)	44 (60.27)	23 (65.71)	0.674
Periodontal pathogens			
* P.g* (*n*, %)	54 (73.97)	33 (94.29)	0.018
* T.d* (*n*, %)	44 (60.27)	29 (82.86)	0.027
* A.a* (*n*, %)	35 (47.95)	24 (68.57)	0.063
Periodontal indicators			
CAL (mm)	3.12 ± 0.69	5.23 ± 0.78	0.000
PD (mm)	4.27 ± 0.43	6.51 ± 0.90	0.000
ABL (%)	22.91 ± 7.04	40.55 ± 9.54	0.000
BOP (%)	47.90 ± 8.97	52.00 ± 10.25	0.036
GI	1.92 ± 0.63	2.17 ± 0.57	0.045
PI	2.14 ± 0.71	2.47 ± 0.78	0.034
Inflammatory Indicators			
IL-6 (pg/mL)	409.69 ± 79.36	450.10 ± 89.54	0.019
IL-8 (pg/mL)	561.13 ± 103.44	604.17 ± 85.65	0.035
TNF (pg/mL)	562.25 ± 153.72	626.19 ± 148.91	0.043

*Abbreviations BMI* body mass index, *PD* probing pocket depth, *CAL* clinical attachment level, *GI* gingival index, *PI* plaque index, *ABL* alveolar bone resorption length, *BOP* bleeding in probing, *IL-6* interleukin-6, *IL-8* interleukin-8, *TNF* tumor necrosis factor, *P.g Porphyromonas gingivalis, T.d*,* Tannerella denticola; A.a*,* Aggregatibacter actinomycetemcomitans*

Subsequently, indicators with significant intergroup differences were incorporated into a binary logistic regression analysis, which identified FAM30A (OR: 9.760, 95%CI: 2.544–37.441, *P* = 0.001), *P.g* (OR: 5.702, 95%CI: 1.070-30.381, *P* = 0.041), ABL (OR: 5.779, 95%CI: 1.659–20.130, *P* = 0.006), and IL-6 (OR: 3.953, 95%CI: 1.085–14.408, *P* = 0.041) as potential risk factors for Stage III/IV periodontitis (Fig. 2B). Furthermore, the ROC curve analysis revealed that FAM30A could predict the occurrence of Stage III/IV periodontitis with a cutoff value of 2.265, yielding a sensitivity of 82.86% and a specificity of 72.60%, and an AUC of 0.841 (95% CI: 0.7633–0.9181, *P* < 0.001, Fig. 2C).

### Reducing FAM30A expression mitigated inflammation triggered by P.g-LPS, improved cell viability, and reduced apoptosis

**Fig. 3:**
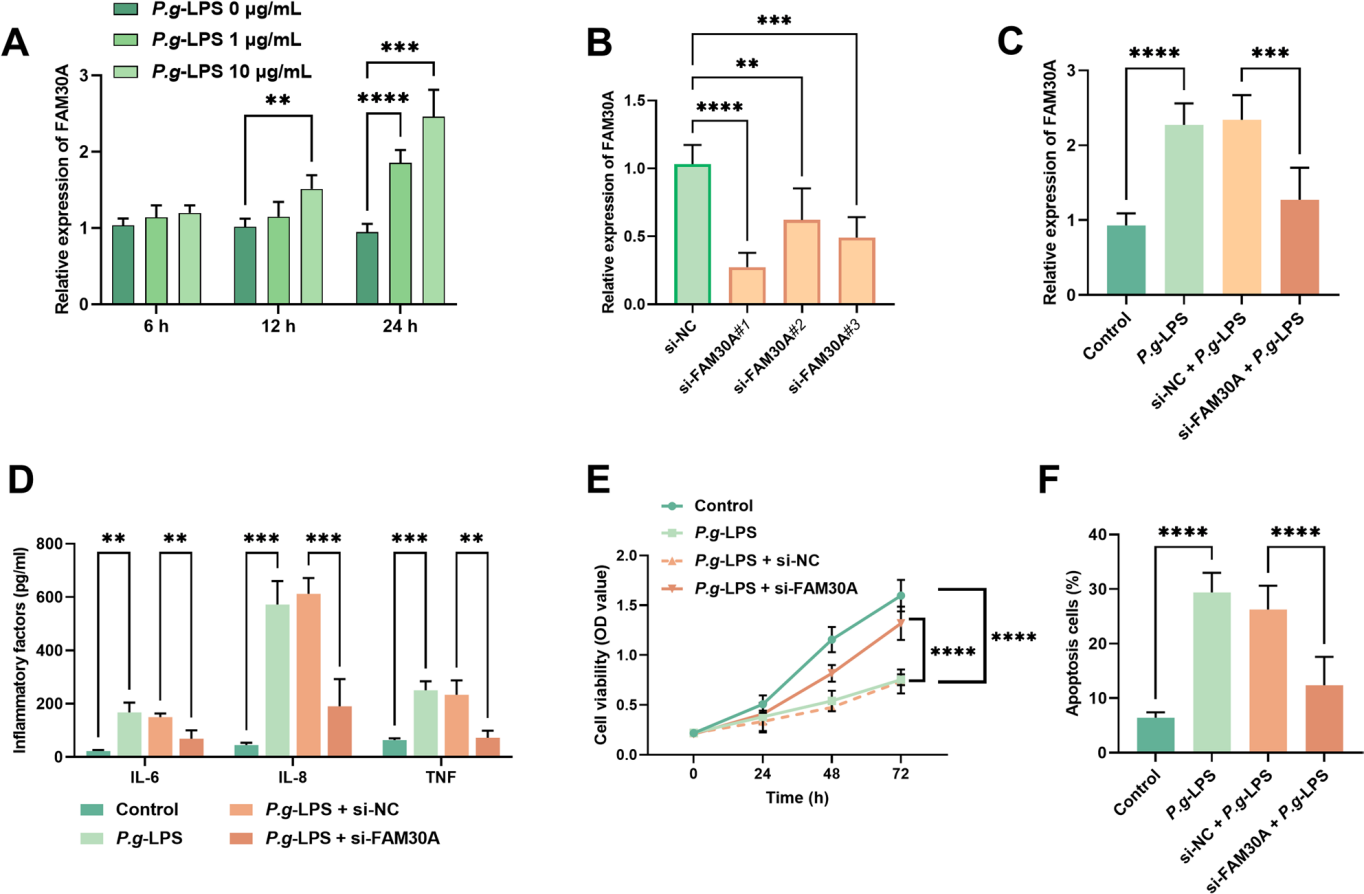
Reducing FAM30A can alleviate *P.g*-LPS-induced inflammation, boosts cell activity, and cut apoptosis (**A**) Effects of different *P.g*-LPS induction times and doses on hPDLCs’ FAM30A expression. **B** Influence of diverse FAM30A siRNA target sequences on intracellular FAM30A. **C** RT-qPCR evaluates FAM30A in hPDLCs following si-FAM30A transfection and *P.g*-LPS induction. **D** ELISA measures inflammatory cytokine levels in cell culture supernatant. **E** CCK-8 analysis of alterations in hPDLCs viability. **F** Flow cytometry detection of apoptosis rates. ** *P* < 0.01; *** *P* < 0.001; **** *P* < 0.0001

To delve deeper into FAM30A’s potential function in periodontitis, we established an in vitro cell model by treating hPDLCs with *P.g*-LPS. As illustrated in Fig. 3A, FAM30A expression in hPDLCs increased in a dose-and time-dependent manner after *P.g-*LPS induction (*P* < 0.001). Based on these results and previous studies, we chose to induce hPDLCs with 10 µg/mL *P.g*-LPS for a 24 h in subsequent experiments. Moreover, compared with the si-NC, different si-FAM30A constructs all effectively decreased FAM30A expression in hPDLCs, with si-FAM30A#1 showing the most significant decrease (*P* < 0.01, Fig. 3B). Thus, si-FAM30A#1 was selected for subsequent experiments. Additionally, si-FAM30A significantly reversed the *P.g-*LPS-induced upregulation of FAM30A expression (*P* < 0.01, Fig. 3C). Moreover, *P.g*-LPS markedly enhanced the secretion of inflammatory cytokines IL-6, IL-8, and TNF in hPDLCs, and this effect was notably diminished by FAM30 silencing (*P* < 0.001, Fig. 3D). Additionally, *P.g*-LPS also significantly suppressed hPDLCs’ viability and induced apoptosis (*P* < 0.001); however, reducing FAM30A expression partially mitigated these *P.g*-LPS-induced effects, leading in partial restoration of cell viability and inhibition of apoptosis (*P* < 0.001, Fig. 3E-F). In summary, silencing FAM30A partially alleviated the damage to periodontal ligament cells.

### Impact of FAM30A on osteogenic differentiation and MMP-1 and MMP-3 regulation in hPDLCs

**Fig. 4:**
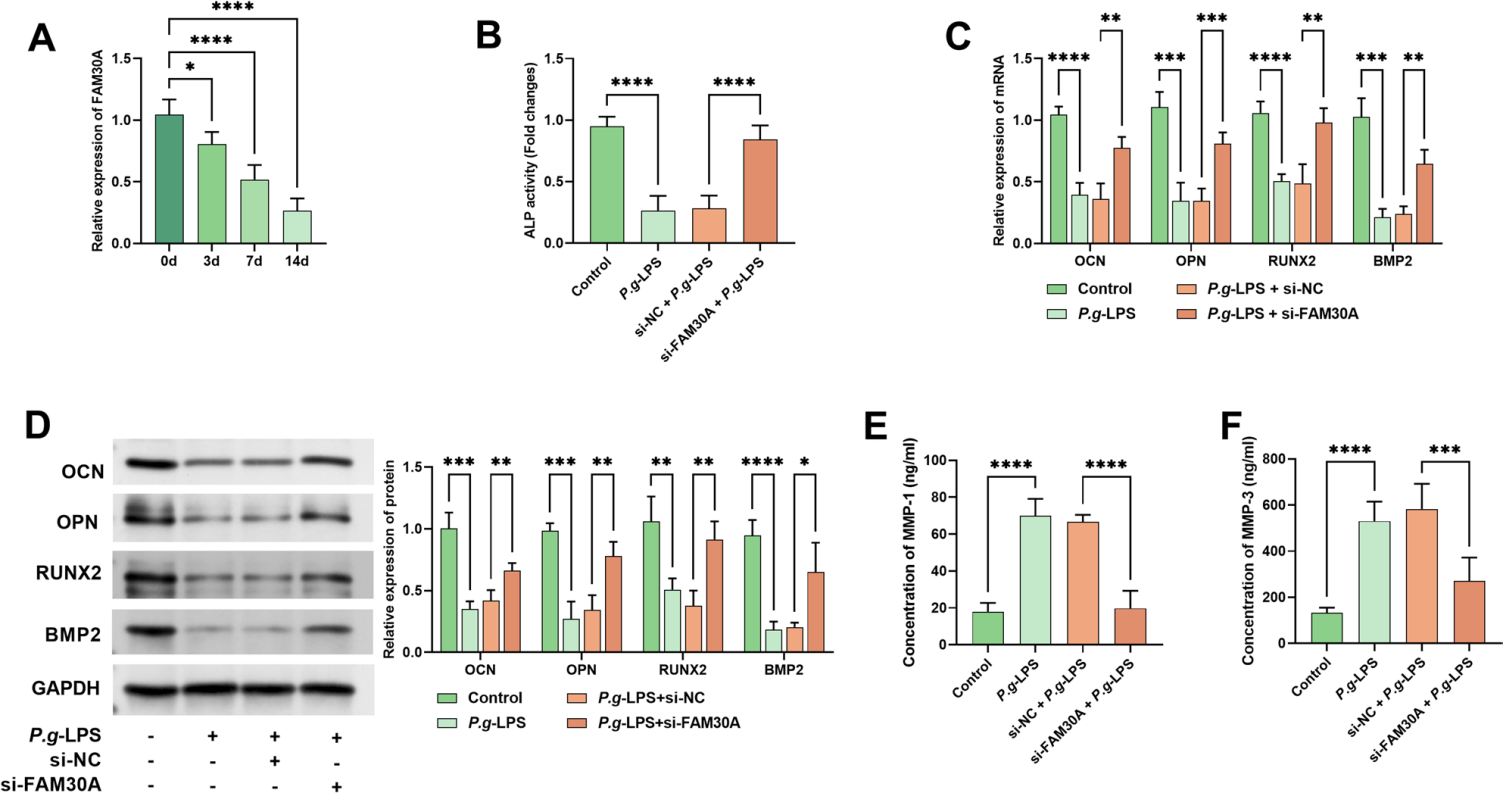
FAM30A suppression boosts osteogenic differentiation and reduces MMP-1/MMP-3 in hPDLCs. **A** FAM30A in hPDLCs drooped time-dependently during osteogenic culture. **B** At day 7 of osteogenic induction, *P.g*-LPS suppressed ALP activity, and si-FAM30A restored it. **C-D** FAM30A silencing partly reversed *P.g*-LPS-induced osteogenic markers downregulation in mRNA and protein expression. **E-F** *P.g*-LPS enhanced MMP-1/MMP-3 secretion in hPDLCs; si-FMA30A weakened this effect. ** *P* < 0.01; *** *P* < 0.001; **** *P* < 0.0001

Figure 4A demonstrates that FAM30A expression in hPDLCs progressively declined during osteogenic induced (*P* < 0.05, Fig. 4A). Notably, *P.g*-LPS boosted FAM30A expression, but si-FAM30A transfection after 7-day osteogenic induction still notably reduced it (*P* < 0.01, Supplementary Figure S3A). *P.g*-LPS suppressed ALP activity, an effect significantly reversed by FAM30A knockdown (*P* < 0.001, Fig. 4B). Similarly, si-FAM30A partially alleviated *P.g*-LPS-mediated downregulation of osteogenic markers (OCN, OPM, RUXN2, and BMP2) at the mRNA and protein levels (*P* < 0.01, Fig. 4C-D, Supplementary Figure S1). Furthermore, *P.g*-LPS-induced MMP-1 and MMP-3 secretion were markedly attenuated by FM30A silencing (*P* < 0.01, Fig. 4E-F).

#### FAM30A was a miR-28-5p sponge

**Fig. 5:**
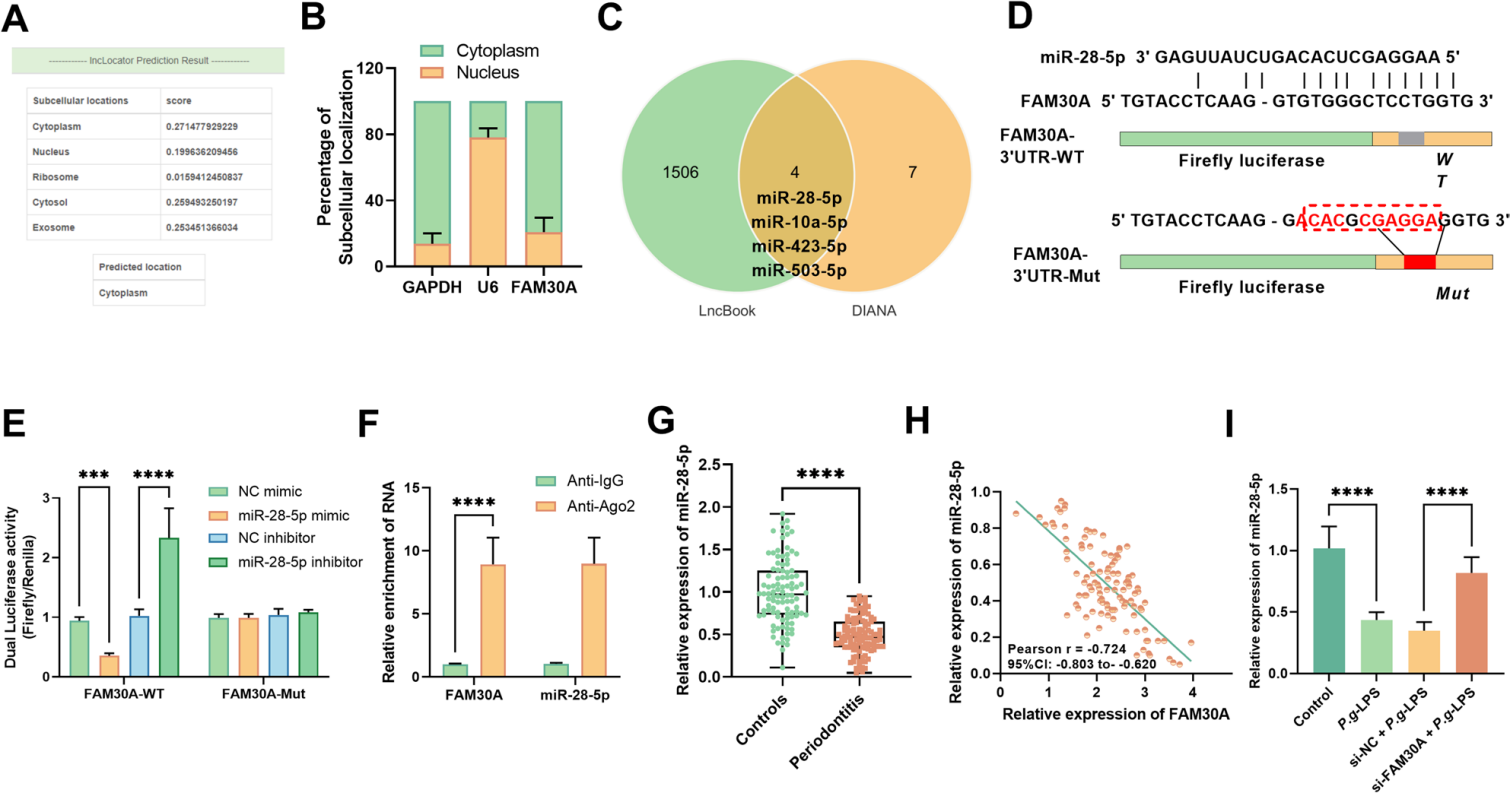
FAM30A was a miR-28-5p sponge. **A** LncLocator database revealed FAM30A mainly in cytoplasm. **B** Subcellular fractionation analyzed FAM30A in hPDLCs. **C** LncBook and DIANA databases co-predicted FAM30A’s candidate miRNA. **D** Displayed binding sequences of FAM30A and miR-28-5p. DLR assays (**E**) and RIP assay (**F)** examined FAM30A and miR-28-5p targeting. **G** miR-28-5p expression in periodontitis patients and controls. **H** Pearson correlation analysis between FAM30A and miR-28-5p. **I** RT-qPCR assessed *P.g*-LPS induction and si-FAM30A transfection levels for the miR-28-5p. ** *P* < 0.01; *** *P* < 0.001; **** *P* < 0.0001

The functionality of lncRNA is associated with its subcellular distribution. Predictions from the LncLocator database revealed that FAM30A is predominantly located in the cytoplasm (Fig. 5A), which was validated by our subcellular fractionation analysis in hPDLCs (Fig. 5B). This cytoplasmic localization pattern implies potential miRNA-targeting mechanisms. Among candidate miRNAs co-predicted by lncBook and DIANA databases, miR-28-5p was chosen for further investigation due to its established relevance in periodontitis (Fig. 5C). The complementary binding sequences between FAM30A and miR-28-5p are illustrated in Fig. 5D. DLR assays demonstrated that miR-28-5p mimic markedly reduced the activity of FAM30A-WT, but had no impact on its mutant form (FAM30A-Mut, *P* > 0.05, Fig. 5E). Furthermore, RIP assay confirmed that both FAM30A and miR-28-5p were preferentially enriched in complexes containing Ago2 compared to IgG controls (*P* < 0.001, Fig. 5F), supporting their direct interaction. In GCF from periodontitis patients, miR-28-5p levels were decreased and inversely correlated with FAM30A expression (*r* = -0.724, *P* < 0.001, Fig. 5G-H). Furthermore, *P.g*-LPS treatment suppressed miR-28-5p expression in hPDLCs, and this effect was notably mitigated by FAM30A knockdown (*P* < 0.001, Fig. 5I).

### MiR-28-5p depletion reverses FAM30A silencing-mediated protection against periodontal ligament injury and osteogenic promotion

**Fig. 6:**
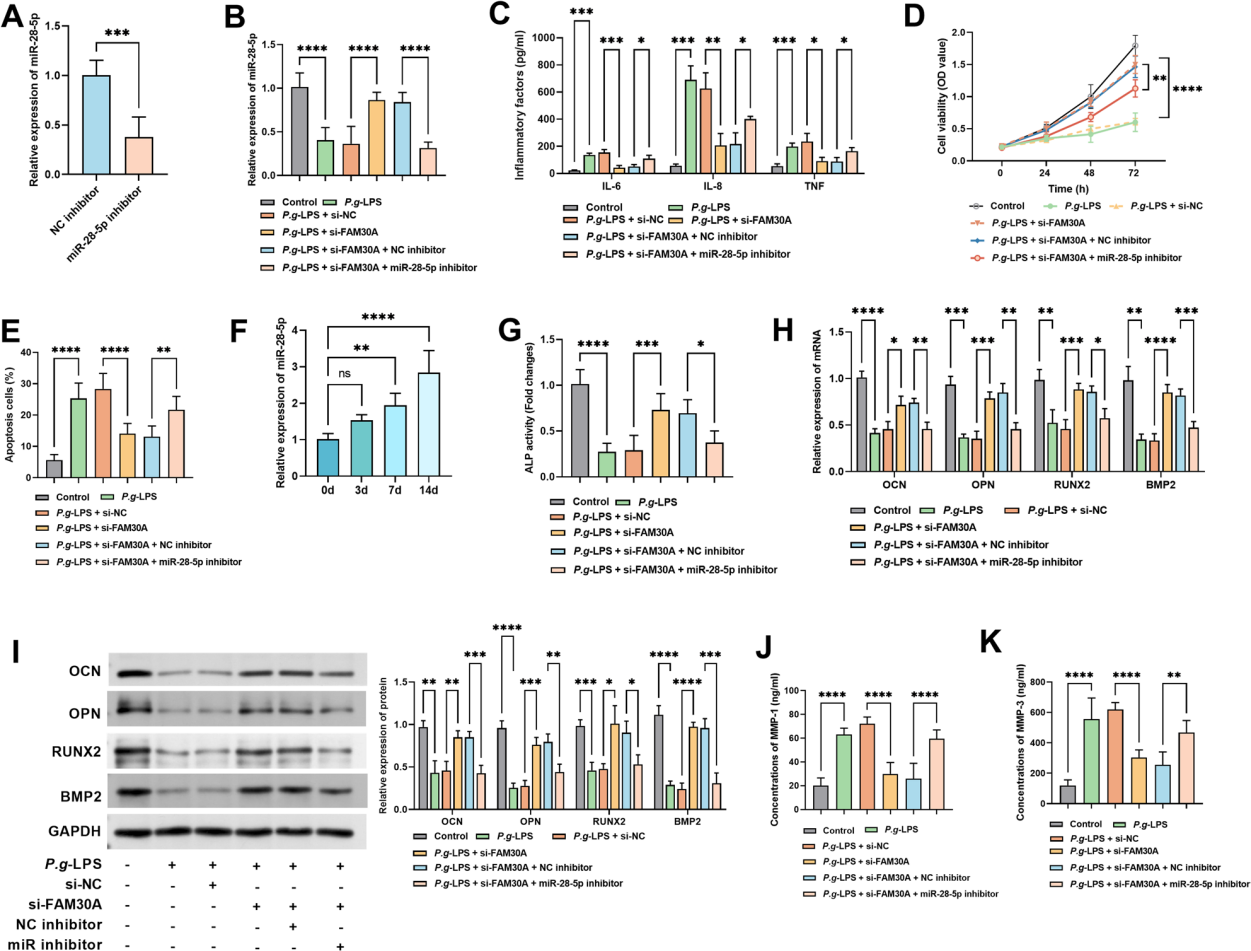
MiR-28-5p depletion reverses FAM30A silencing’s protective and osteogenic effects on periodontal ligaments. **A-B** RT-qPCR assessed miR-28-5p levels in hPDLCs with miR-28-5p transfection, *P.g*-LPS induction, and si-FAM30A transfection. **C** ELISA measured cellular inflammatory factor secretion after miR-28-5p reduction. **D-E** CCK-8 and flow cytometry analyzed cell viability and apoptosis after miR-28-5p lowering. **F** Examined miR-28-5p levels at different osteogenic differentiation induction times. **G-I** Measured ALP activity and osteogenic markers mRNA and protein expression at various induction time and on day 7. **J-K** ELISA analyzed MMP-1 and MMP-3 levels in hPDLCs. * *P* < 0.05; ** *P* < 0.01; *** *P* < 0.001; **** *P* < 0.0001

To investigate the role of FAM30A in the progression of periodontitis through miR-28-5p regulation, we transfected hPDLCs with a miR-28-5p inhibitor. This inhibitor successfully lowered miR-28-5p levels (*P* < 0.05, Fig. 6A) and attenuated si-FAM30A-triggered increase in miR-28-5p under *P.g*-LPS stimulation (*P* < 0.05, Fig. 6B). Functionally, depleting miR-28-5p partially counteracted the inhibitory effect si-FAM30A on the secretion of inflammatory cytokine while abolishing its pro-survival (enhanced cell viability) and anti-apoptotic effects in hPDLCs (*P* < 0.05, Fig. 6C-E). During osteogenic induction, miR-28-5p expression increased time dependently in hPDLCs (*P* < 0.05, Fig. 6F). *P.g*-LPS notably repressed miR-28-5p expression. By day 7 of osteogenic differentiation induction, this repression was significantly alleviated, yet such relief was substantially diminished upon miR-28-5p inhibitor (*P* < 0.01, Supplementary Figure S3B). On day 7, si-FAM30A significantly enhanced ALP activity and osteogenic marker (OCN, OPN, RUNX2) mRNA and protein expression while suppressing MMP-1 and MMP-3 secretion (*P* < 0.05, Fig. 6G-K, Supplementary Figure S2). Notably, these effects were substantially reversed by miR-28-5p inhibition (*P* < 0.05, Fig. 6G-K).

### MiR-28-5p Targets the 3’UTR of KAT6A

**Fig. 7:**
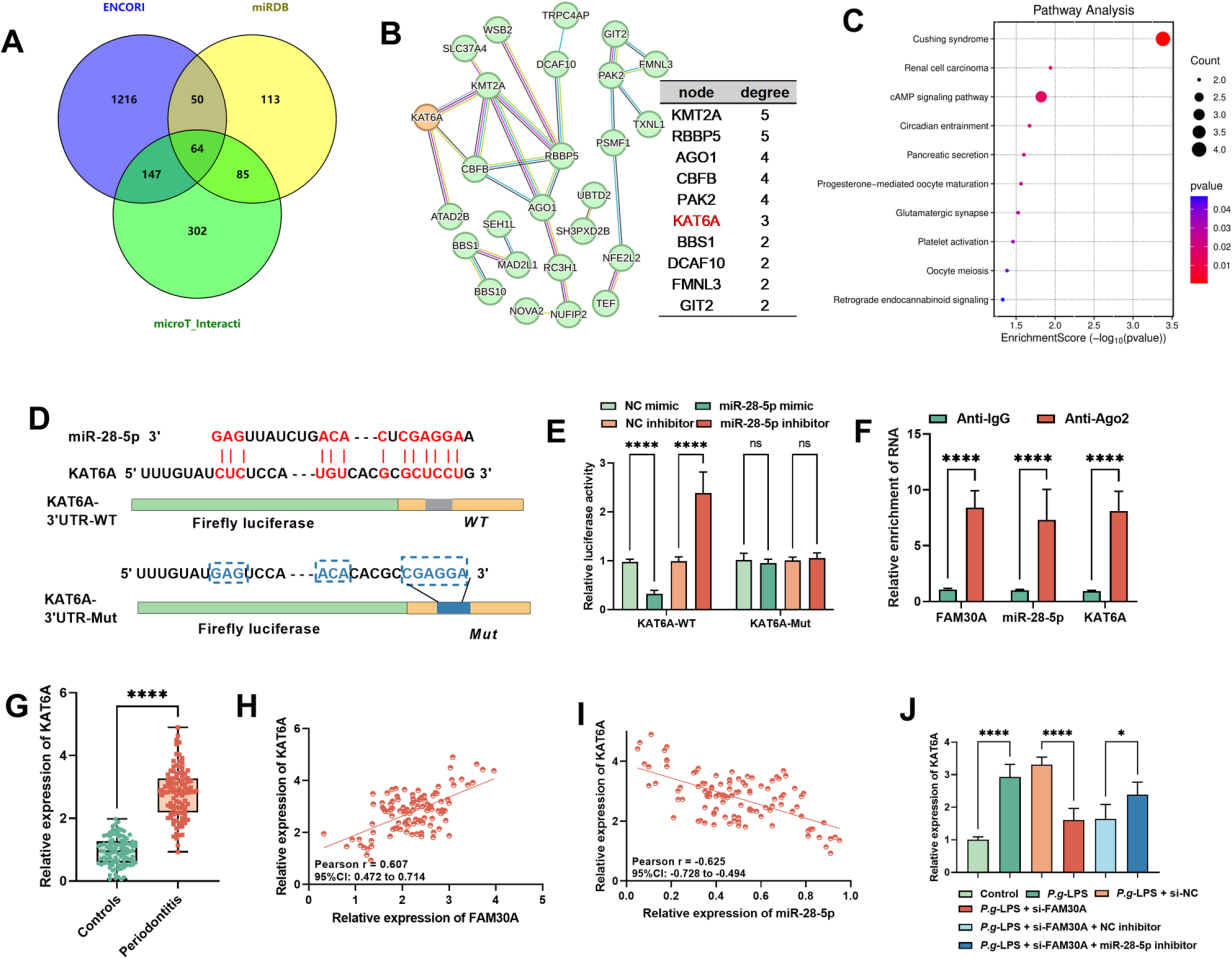
MiR-28-5p Targets the 3’UTR of KAT6A. **A** A Venn diagram displays genes predicted as miR-28-5p targets, overlapping across three databases. **B** PPI network illustrates overlapping target genes and highlights the top 10 hub genes. **C** KEGG analysis identified signaling pathways enriched among the overlapping targets. **D** Predicted binding site for miR-28-5p on KAT6A. **E-F** DLR and RIP assays confirmed the targeted interaction between KAT6A and miR-28-5p. G. RT-qPCR measured serum KAT6A expression levels. **H-I** Pearson correlation analysis assessed the relationships between serum KAT6A levels and serum FAM30A or miR-28-5p in periodontitis patients. **J** *P.g*-LPS modulated FAM30A and miR-28-5p expression in hPDLCs, influencing KAT6A levels. * *P* < 0.05; *** *P* < 0.001; **** *P* < 0.0001; ns, not significant

To delve deeper into how the FAM30A/miR-28-5p axis drives periodontitis progression, we examined its downstream mRNAs. Figure 7A reveals that 64 overlapping target genes were pinpointed from the ENCORI, miRDB, and microT_interaction databases. These genes formed a PPI network with 64 nodes (representing genes) and 27 edges (indicating relationships), yielding a PPI enrichment p-value of 0.00045 (Fig. 7B). Additionally, 10 hub genes, including KAT6A, were identified. KEGG pathway enrichment analysis revealed that these overlapping targets were primarily enriched in the cAMP signaling pathway (Fig. 7C). Figure 7D depicted the targeted binding sites between KAT6A and miR-28-5p. DLR analysis demonstrated that the miR-28-5p mimic notably decreased luciferase activity in KAT6A-WT, without affecting KAT6A-Mut activity (Fig. 7E). Additionally, FAM30A, miR-28-5p, and KAT6A all exhibited enrichment on Ago2 antibodies compared to IgG (*P* < 0.001, Fig. 7F). Furthermore, compared to the controls, periodontitis patients had elevated serum KAT6A levels (*P* < 0.001, Fig. 7G). Moreover, these levels in periodontitis patients were significantly positively correlated with FAM30A (*r* = 0.607, *P* < 0.001, Fig. 7H) and negatively correlated with miR-28-5p (*r* = -0.625, *P* < 0.001, Fig. 7I). Finally, *P.g*-LPS notably increased KAT6A levels in hPDLCs, an effect markedly reduced by FAM30A inhibition and partially reversed by miR-28-5p inhibitor (*P* < 0.001, Fig. 7J).

## Discussion

**Fig. 8:**
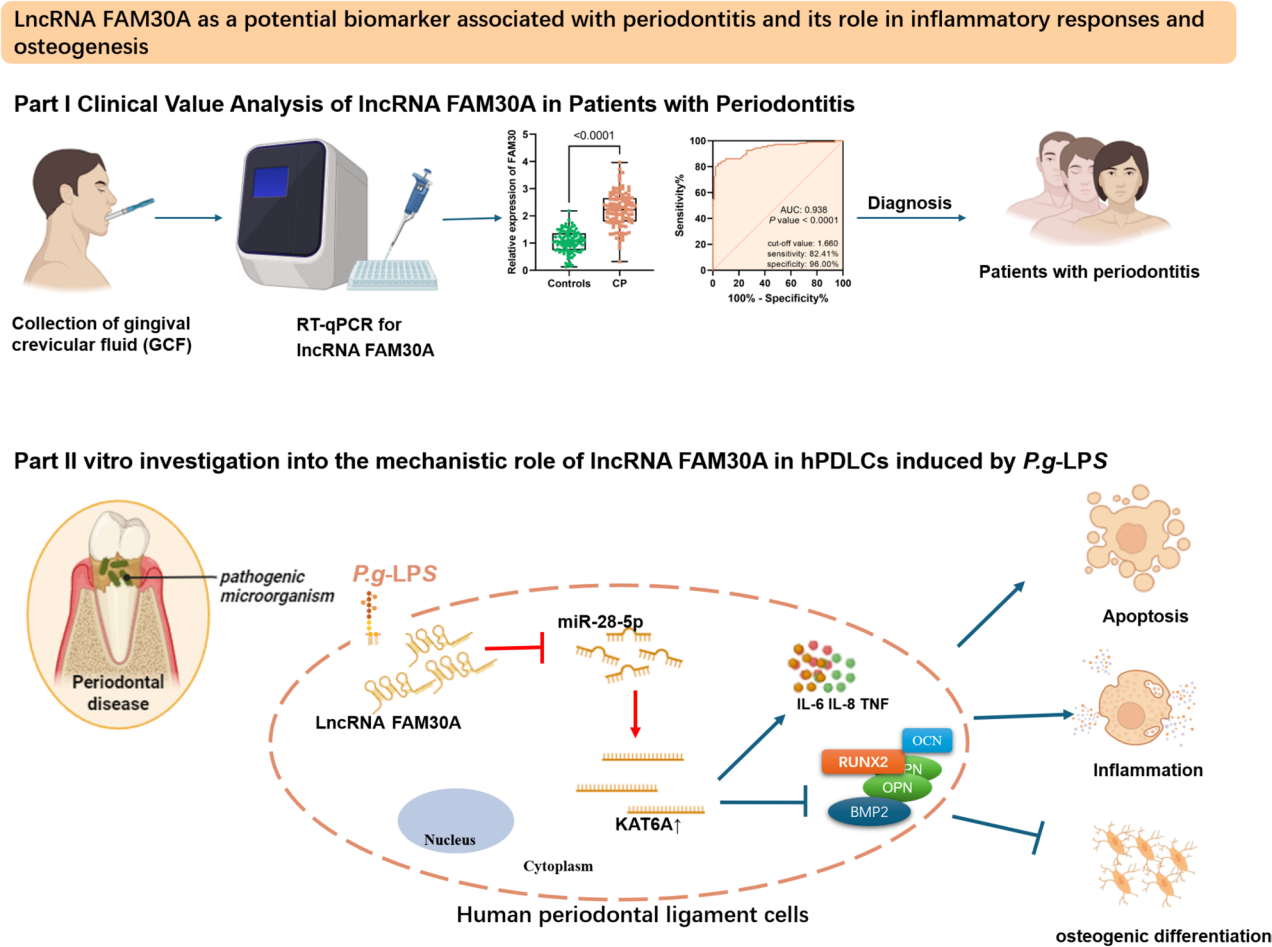
Graphical summary of this study. This study demonstrates that, in clinical settings, markedly increased FAM30A levels in the gingival crevicular fluid (GCF) of periodontitis patients underscore its diagnostic promise for periodontitis. In vitro experiments indicate that P.g-LPS triggers inflammatory reactions and apoptosis in human periodontal ligament cells (hPDLCs), while concurrently suppressing their osteogenic differentiation potential, through modulation of the lncRNA FAM30A/miR-28-5p/KAT6A signaling axis

Previous studies have highlighted the clinical potential of lncRNAs in periodontitis and their mechanisms of action. Notably, Wu et al. reported a 2.33-fold upregulation of FAM30A by analyzing differentially expressed lncRNAs in periodontitis [5]. Similarly, our analysis of the GSE10334 dataset revealed a 2.01-fold increase in FAM30A expression in periodontitis patients. However, the precise role and underlying mechanisms of FAM30A in periodontitis remain unclear. In this study, we found that FAM30A levels were significantly elevated in the GCFs of patients with periodontitis, highlighting its diagnostic value in distinguishing periodontitis from periodontal health. FAM30A levels also correlated with disease staging, which helps in assessing patients’ conditions. Moreover, downregulating FAM30A reversed the adverse effects of *P.g*-LPS, such as cellular inflammation, reduced viability, increased apoptosis, and inhibited osteogenic differentiation in hPDLCs (Fig. 8).

In periodontology, researchers aim to understand periodontitis pathogenesis and find better diagnostic and treatment methods. Prior research has mainly highlighted the buildup and Gram-negative bacteria in dental plaque, especially the crucial role of specific bacteria like *P.g*,* T.d*, and *A.a* in periodontitis onset [22]. Yet, it should be noted that periodontitis development isn’t solely reliant on these bacteria; it can still emerge without them. In our study, among periodontitis patients, those with higher FAM30A expression had higher positive culture rates for key pathogens such as the above-mentioned bacteria. These findings support the notion that FAM30A is likely closely linked to the colonization or infection process of these major pathogens. It further suggests that FAM30A may be involved in the initiation of periodontitis by affecting its pathogenic potential, thereby offering novel insights into the pathogenesis of this disease. Additionally, our research revealed that elevated FAM30A expressions were correlated with higher values of periodontal clinical parameters, including CAL, PD, ABL, BOP, and PI. These results substantiate that FAM30A is strongly associated with the staging of periodontitis and various critical clinical manifestations, making it a promising biomarker for evaluating the progression of periodontitis. This was further validated in subsequent research, where patients with stage III/IV periodontitis exhibited increased GCF’s FAM30A levels. Moreover, we identified FAM30A, in conjunction with *P.g*, IL-6, and ABL, as potential risk factors for disease progression and deterioration. Meanwhile, we discovered that FAM30A can distinguish individuals with periodontitis from healthy subjects and assess the staging of the disease. This endows FAM30A with immense clinical significance in the diagnosis and treatment of periodontitis. It is poised to become a novel, efficient tool for diagnosing and monitoring periodontitis, bridging the gap in current diagnostic methods. It will strongly support early detection, intervention, and precise treatment of periodontitis, thereby enhancing oral health and the quality of life for patients.

Periodontitis manifests as gingival recession, alveolar bone resorption, periodontal ligament degradation, and dental caries, accompanied by gum swelling, bleeding, inflammatory cell infiltration, and ultimately, tooth loss [23]. Previous research has indicated that the key pathology of periodontitis involves causative agents such as *P.g* -LPS triggering an inflammatory response in PDLCs [24]. This, consequently, impairs cell viability, disrupts osteogenic function, and degrades the periodontal tissue matrix. Previous studies have revealed that LncRNA NR_045147 participates in the osteogenic differentiation and migration of PDLCs [25]. Overexpression of TUG1 in PDLCs mitigates LPS-induced suppression of proliferation and promotion of apoptosis [26]. Accumulating evidence underscores the pivotal role of FAM30A in the progression of various inflammatory conditions. For instance, a network analysis-based study demonstrated its involvement in the pathology of lupus nephritis pathology and its potential as a liquid biopsy biomarker [13]. Another study identified FAM30A as a promising therapeutic target for rheumatoid arthritis [14]. These insights collectively imply that FAM30A may also be play a crucial role in the pathogenesis of periodontitis. In our study, we found that PDLCs exposed to *P.g*-LPS overproduced inflammatory factors, a phenomenon that was markedly reduced when FAM30A expression was low. This indicates that FAM30A likely plays a crucial regulatory role in the onset of periodontitis, offering a novel potential target for deepening our understanding of its pathogenesis. PDLCs, a heterogeneous cell population derived from periodontal tissues, can proliferate to differentiate into fibroblasts, osteoblasts, and osteocytes. These cells are intimately involved in the reconstruction and regeneration of periodontal defects [27]. We discovered that lowering FAM30A levels mitigated the *P.g*-LPS-induced suppression of hPDLC proliferation and the increase in apoptosis, thereby preventing PDLC cellular dysfunction. Osteogenic differentiation of hPDLCs is pivotal for periodontal tissue regeneration; however, it is significantly impaired in an inflammatory microenvironment. Fortunately, our research revealed that silencing FAM30A could counteract the inhibitory effect of *P.g*-LPS on hPDLC osteogenic differentiation, indicating the significant role of FAM30A in regulating this process and promoting periodontal tissue regeneration. Additionally, we thoroughly investigated alterations in other relevant molecules during periodontitis pathogenesis. We observed that *P.g*-LPS stimulation markedly elevated the levels of MMP-1 and MMP-3. These proteases contribute significantly to periodontal tissue degradation by breaking down its extracellular matrix, thereby worsening tissue damage [28]. However, suppressing FAM30A expression resulted in a notable decrease in the secretion of MMP-1 and MMP-3 secretion. This finding further implies that FAM30A may be participate in the inflammatory response and tissue destruction of periodontitis by modulating the secretion of MMP-1 and MMP-3. In summary, FAM30A plays a multifaceted and pivotal role in the progression of periodontitis and the repair/regeneration of periodontal tissue. It accomplishes this by regulating inflammatory responses, cell proliferation, apoptosis, osteogenic differentiation, and the secretion of related proteases (such as MMP-1 and MMP-3) in hPDLCs. This discovery offers fresh insights into periodontitis pathogenesis and provides a novel potential target and theoretical foundation for treating periodontitis, potentially driving innovation in periodontitis therapies.

Previous research has demonstrated that cytoplasmic lncRNAs can function as molecular sponges for miRNAs, thereby influencing disease progression. Our database analysis and sub-cellular localization studies in hPDLCs reveal that these lncRNAs are predominantly located in the cytoplasm, implying their potential to act as miRNA sponges. Notably, prior studies have demonstrated that FAM30A may contribute to the progression of colorectal cancer by modulating the levels of miR-21-3p. Given this, we aimed to identify potential miRNAs that FAM30A might target to influence the pathogenesis of periodontitis. Among numerous miRNAs, miR-28-5p was found to be significantly downregulated in periodontitis patients and to be involved in regulating inflammatory factor expression [29]. A miRNA microarray analysis comparing 44 periodontitis patients with controls also confirmed the marked reduction of miR-28-5p in periodontitis patients [30]. Moreover, abnormal miR-28-5p expression was observed in periodontitis associated with oral *Fusobacterium nucleatum* infection [31]. In line with prior research, we observed a notable downregulation of miR-28-5p in the GCFs of patients with periodontitis. Previous studies have also demonstrated a progressive rise in miR-28 during the osteogenic differentiation of human bone marrow mesenchymal stem cells [32]. Additionally, miR-28-5p has been reported to be involved in the osteogenic differentiation of human exfoliated deciduous tooth MSCs by modulating the interaction with signal transducer and activator of transcription 1 (STAT1) mRNAs [33]. Consistent with these findings, the levels of miR-28-5p in hPDLCs gradually declined with prolonged osteogenic induction. Notably, we made a novel discovery that FAM30A can function as a sponge for miR-28-5p. Moreover, reducing the levels of miR-28-5p effectively counteracted the effects of FAM30A downregulation on *P.g*-LPS-induced inflammation, apoptosis, and the levels of MMP-1 and MMP-3.

KAT6A, a histone lysine acetyltransferase also termed MOZ or MYST3, is a member of the MYST family. Prior research has explored its possible involvement in breast cancer [34] and acute myeloid leukemia [35]. Recent research has revealed KAT6A’s involvement in the stemness and osteogenic differentiation of human bone marrow mesenchymal stem cells. Notably, KAT6A mutations are linked to bone marrow failure [36]. Additionally, KAT6A modulates osteoclast genesis in orthodontically treated teeth [37]. Highly expressed in periodontal-derived cells, KAT6A promotes osteogenic differentiation by regulating histone acetylation and osteogenesis-related gene expression [38]. Moreover, KAT6A levels are elevated in periodontitis patients, and its deficiency reduces inflammatory macrophage response [39]. In line with prior research, our study observed notably higher KAT6A levels in periodontitis patients. We are the first to show that KAT6A is a target of the FAM30A/miR-28-5p axis, with FAM30A upregulation and miR-28-5p downregulating its expression. Notably, miR-28-5p targets were mainly enriched in the cAMP signaling pathway. Previous studies have indicated that LPS-stimulated human gingival fibroblasts show significantly increased intracellular cAMP levels [40]. Additionally, odontoblasts possess an extracellular Ca^2+^-sensing mechanism, with the cAMP pathway playing a pivotal role in periodontal bone formation [41]. However, further exploration is needed to determine whether the FAM30A/miR-28-5p and KAT6A axis contribute to periodontitis progression via the cAMP pathway.

This study is the first to uncover the aberrant expression profile of lncRNA FAM30A in periodontitis and validate its clinical potential as a diagnostic biomarker for the disease. Stratified analysis of clinical samples revealed that FAM30A is strongly associated with periodontitis staging and can effectively distinguish between different stages of periodontitis. This offers new molecular evidence for the clinical stratification of patients and the formulation of personalized treatment plans. This finding bridges a knowledge gap in research on FAM30A as a diagnostic biomarker for periodontitis. Compared with transitional clinical biomarkers, FAM30A might demonstrate greater specificity and sensitivity, presenting a novel technical approach for early disease screening and ongoing condition monitoring. This study is the pioneer in clarifying the molecular mechanism where FAM30A targets and modulates the miR-28-5p/KAT6A axis via a ceRNA mechanism, leading to over-activation of inflammatory responses and suppression of periodontal bone formation. Previous research has mainly centered on either the inflammatory pathway in periodontitis or the isolated functions of bone metabolism-related genes. In contrast, this study is the first to link the lncRNA-miR-28-5p axis with the pathological course of periodontitis, uncovering its central regulatory function. It offers a new molecular insight into the pathophysiology of periodontitis and establishes a theoretical basis for creating targeted therapeutic strategies. Moreover, building on the above-mentioned clinical value and mechanism investigations, this study suggests that targeted inhibition of FAM30A and miR-28-5p could be a promising therapeutic approach to ease periodontitis. It offers fresh insights and experimental support for targeted treatment of the disease.

This study has limitations. First, though we strictly used inclusion/ exclusion criteria and excluded patients with autoimmune, endocrine, and systemic chronic inflammatory diseases, we overlooked mild hypertension and obesity in some cases. Second, socioeconomic status, covering income, education, and occupation, makes data collection tough and assessment complex. So, we initially excluded it. But it can indirectly impact oral health. Hence, in future studies with larger samples and deeper analyses, we’ll control for this factor and perform in-depth quantitative analysis of bacterial load in patient samples. Furthermore, periodontitis stems from polymicrobial causes. Here, we used *P.g*-LPS to induce hPDLCs, building an in vitro periodontitis model. This common method isn’t comprehensive, a study limitation. We’ll validate findings with multi-microbe-induced PDLCs and gingival cells later. What’s more, in our in vitro cell studies, we assessed ALP activity and the mRNA/protein expression of osteogenic differentiation markers. However, the lack of alizarin red staining is an additional potential limitation, which we plan to resolve in future studies. Furthermore, although our current study has focused on analyzing the roles of FAM30A/miR-28-5p/KAT6A axis in hPDLCs, the function of these molecules in gingival cells remain an area that warrants in-depth further exploration.

In summary, our comprehensive analysis identify FAM30A, for the first time, as a promising diagnostic biomarker for periodontitis, with its levels closely correlated with disease staging. Inhibiting FAM30A could potentially decelerate periodontitis progression by modulating miR-28-5p/KAT6A axis, thereby dampening inflammation and fostering bone regeneration.

## Supplementary Information

Supplementary Material 1.

## Data Availability

The data that support the findings of this study are not publicly available due to privacy reasons, but are available from the corresponding author upon reasonable request.
