# Supplementary material for: LncRNA FAM30A as a potential biomarker associated with periodontitis and its role in inflammatory responses and osteogenesis
Source: BMC Oral Health. 2026 Mar 13;26:706. doi: 10.1186/s12903-026-08033-y (PMC13097576; doi:10.1186/s12903-026-08033-y)
Supplement: Supplementary file 1 — Supplementary Material 1. [file 12903_2026_8033_MOESM1_ESM.docx]

**Supplemental Table S1** Primer sequences used for PCR

| Gene | Forward primer (5’→3’) | Reverse primer (5’→3’) |
| --- | --- | --- |
| FAM30A | CTGTGGCAAAGGCAAGTGAC | TTTCTTCCCTGTGTGGGGAGTC |
| GAPDH | ATGACATCAAGAAGGTGGTG | CATACCAGGAAATGAGCTTG |
| miR-28-5p | GCGGAAGCTCACAGTCT | TGGTGTCGTGGAGTCG |
| U6 | TGCGGGTGCTCGCTTCGGCAGC | CCAGTGCAGGGTCCGAGGT |
| KAT6A | GAGGCCAATGCCAAGATTAGAAC | GTTGATACTAGAGCCGCTGCCTC |
| OCN | CTGACCTCACAGATCCCAAGC | TGGTCTGATAGCTCGTCACAAG |
| OPN | AGCAAGAAACTCTTCCAAGCAA | GTGAGATTCGTCAGATTCATCCG |
| RUNX2 | ATGCTTCATTCGCCTCACAAA | GCACTCACTGACTCGGTTGG |
| BMP2 | GTGCTTCTTAGACGGACTGCGGTCTCCTA | GCCTGAGTGCCTGCGGTACAGATCTAG |
| *P.g* | AGGCAGCTTGCCATACTGCG | ACTGTTAGCAACTACCGATGT |
| *T.d* | TAATACCGAATGTGCTCATTTACAT | TCAAAGAAGCATTCCCTCTTCTTCTTA |
| *A.a* | ATGCCAAATTGACGTTAAAT | AAACCCATCTCTGAGTTCTTCTTC |

**
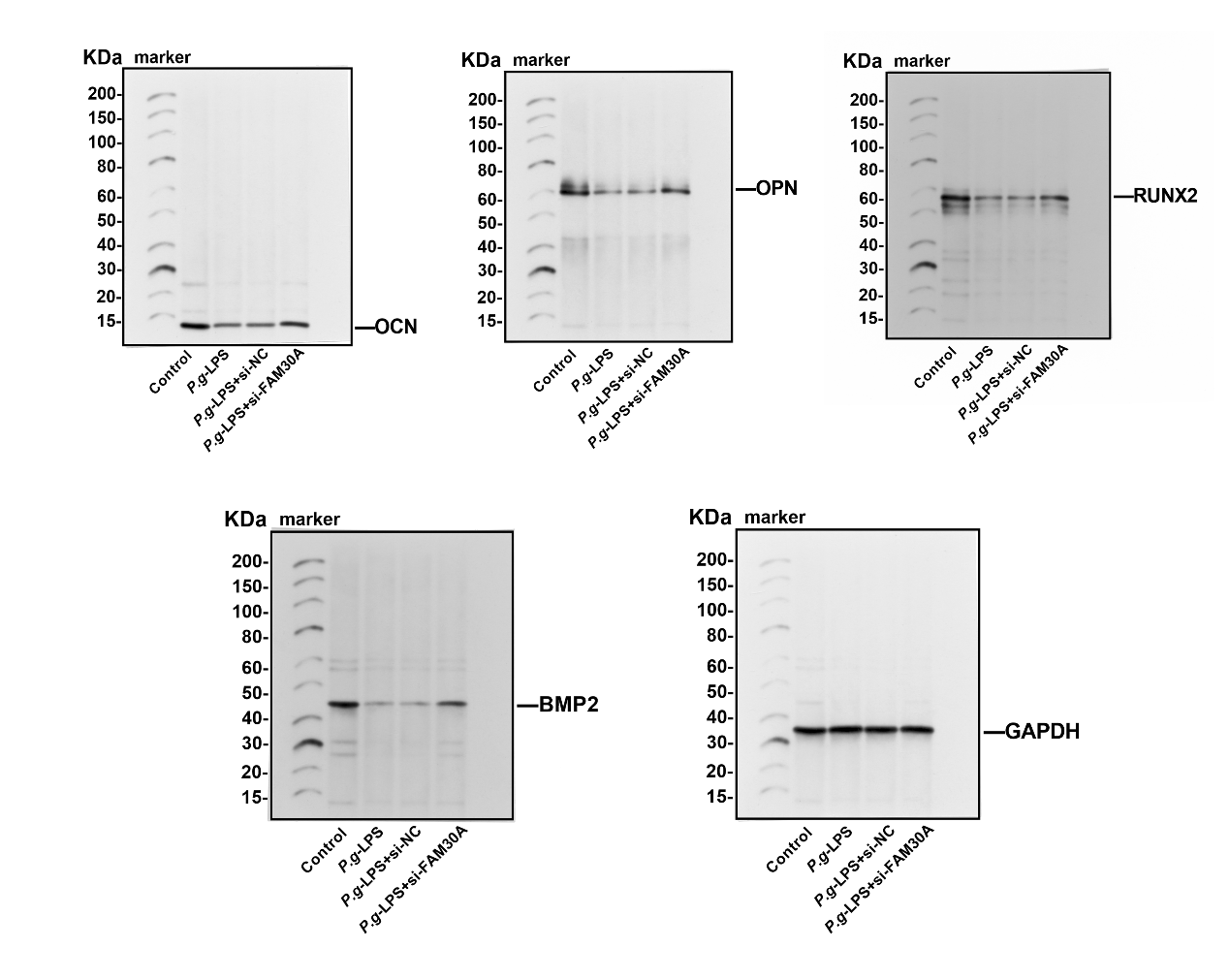
**

**Supplementary Figure S1**. The original uncut full membrane image of Western blot analysis of the expression levels of osteogenic differentiation marker proteins in human periodontal ligament cells induced by *P.g*-LPS after inhibition of FAM30A. The corresponding cropped image is presented in Figure 4D.


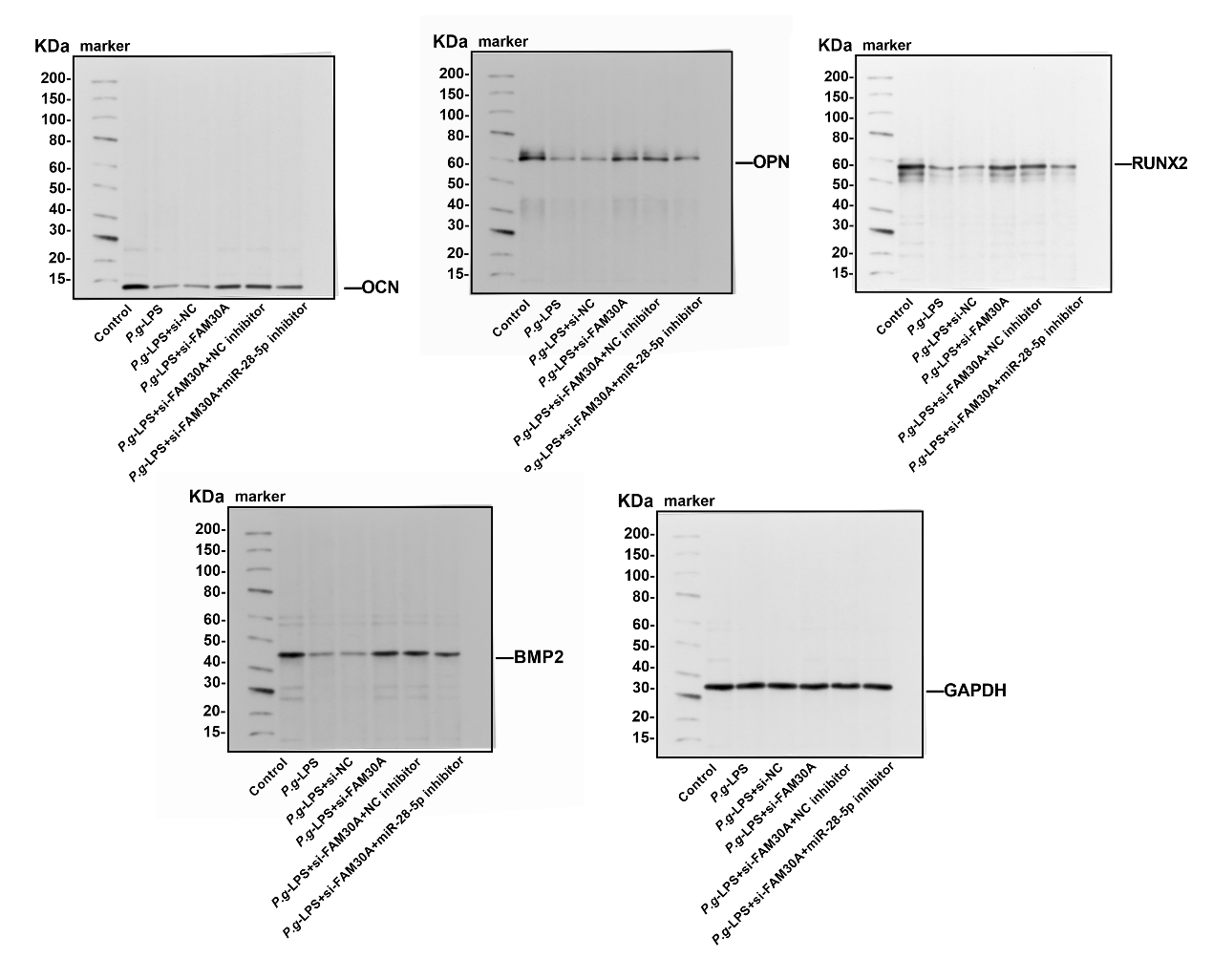


**Supplementary Figure S2**. The original uncut full membrane image of Western blot analysis of the expression levels of osteogenic differentiation marker proteins in human periodontal ligament cells induced by *P.g*-LPS after inhibition of FAM30A and miR-28-5p. The corresponding cropped image is presented in Figure 6I.


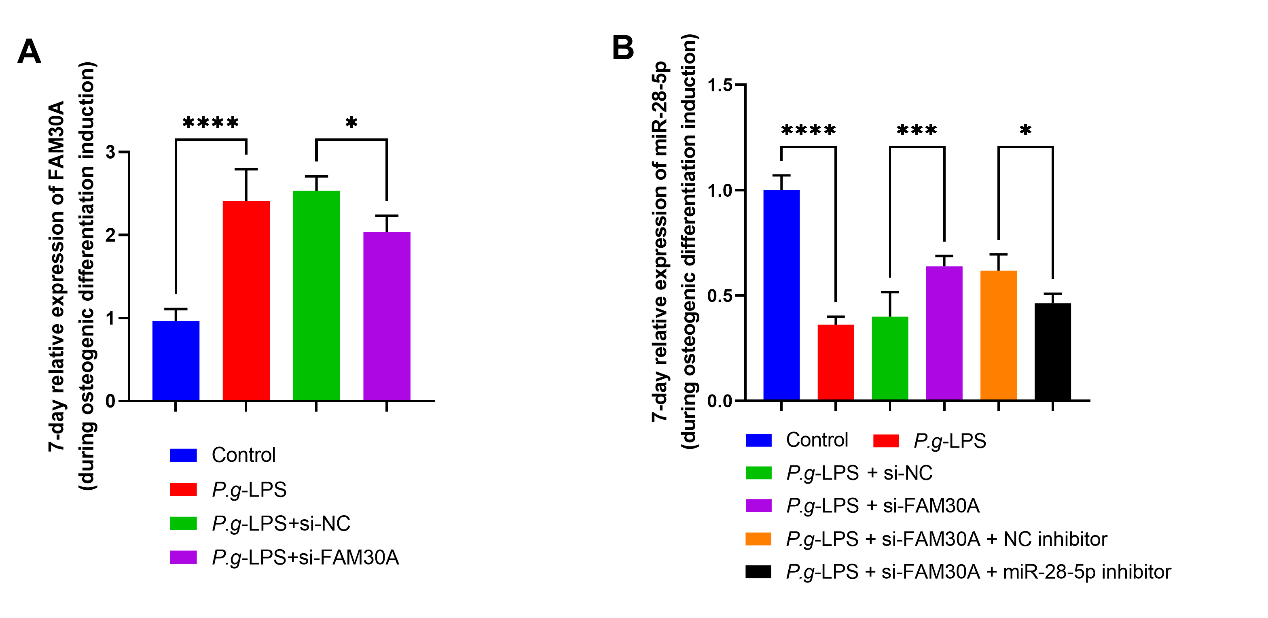


**Supplementary Figure S3.** Impact of si-FAM30A transfection and miR-28-5p inhibitor treatment on FAM30A (A) and miR-28-5p (B) expression levels in hPDLCs during a 7-day osteogenic induction process. * *P* < 0.05, ** *P* < 0.01, **** *P* < 0.0001.
